# Mitosis Gives a Brief Window of Opportunity for a Change in Gene Transcription

**DOI:** 10.1371/journal.pbio.1001914

**Published:** 2014-07-29

**Authors:** Richard P. Halley-Stott, Jerome Jullien, Vincent Pasque, John Gurdon

**Affiliations:** 1 Wellcome Trust/Cancer Research UK Gurdon Institute, The Henry Wellcome Building of Cancer and Developmental Biology, Cambridge, United Kingdom; 2 Department of Zoology, University of Cambridge, Cambridge, United Kingdom; 3 Department of Biological Chemistry, David Geffen School of Medicine, The Eli and Edith Broad Center of Regenerative Medicine and Stem Cell Research, University of California, Los Angeles, California, United States of America; Harvard University, United States of America

## Abstract

Cell differentiation is remarkably stable but can be reversed by somatic cell nuclear transfer, cell fusion, and iPS. Nuclear transfer to amphibian oocytes provides a special opportunity to test transcriptional reprogramming without cell division. We show here that, after nuclear transfer to amphibian oocytes, mitotic chromatin is reprogrammed up to 100 times faster than interphase nuclei. We find that, as cells traverse mitosis, their genes pass through a temporary phase of unusually high responsiveness to oocyte reprogramming factors (mitotic advantage). Mitotic advantage is not explained by nuclear penetration, DNA modifications, histone acetylation, phosphorylation, methylation, nor by salt soluble chromosomal proteins. Our results suggest that histone H2A deubiquitination may account, at least in part, for the acquisition of mitotic advantage. They support the general principle that a temporary access of cytoplasmic factors to genes during mitosis may facilitate somatic cell nuclear reprogramming and the acquisition of new cell fates in normal development.

## Abstract 2

In mitotic nuclei transplant experiments, many genes undergo major changes in gene expression. This supports the idea that mitosis facilitates new cell fate decisions during normal development.

## Author Summary

Cells are dividing very actively at a time in development when new gene expression and new cell lineages arise. At mitosis, most transcription factors are temporarily displaced from chromosomes. We show that, after transplantation to oocytes, somatic cell nuclei that have been synchronized in mitosis can be reprogrammed to pluripotency gene expression up to 100 times faster than interphase nuclei. We find that, as cells traverse mitosis, their genes pass through a temporary phase of unusually high responsiveness to oocyte reprogramming factors (mitotic advantage). Many other genes in the genome have also shown a mitotic advantage, which affects the rate rather than the final level of transcriptional enhancement. This is attributable to a chromatin state rather than to more rapid passage of reprogramming factors through the nuclear membrane. Histone H2A deubiquitination at mitosis is required for the acquisition of mitotic advantage. Our results support the general principle that a temporary access of cytoplasmic factors to genes during mitosis facilitates somatic cell nuclear reprogramming and the acquisition of new cell fates in normal development.

## Introduction

Normal development, as well as nearly all cases of experimentally induced changes in gene transcription, is accompanied by cell division. It is therefore hard to distinguish those molecular events which prepare cells for, or engage them in, mitosis from those that are required specifically for transcriptional reprogramming. The relationship between the cell cycle and cell fate decisions has for a long time attracted interest [1]. Transition through mitosis is a time when many transcription factors are displaced from chromatin, potentially permitting new transcription factors to occupy chromatin sites on mitotic exit and so direct a postmitotic cell fate change [2]–[5]. Mitotic remodelling has been shown to be of great importance for the efficient replication of erythrocyte nuclei by *Xenopus* egg extracts [6],[7]. For new transcription, cell division seems to be needed in some cases [8],[9] but not in others [10],[11]. Here we have used nuclear transfer to amphibian oocytes to compare directly the ability of mitotic chromatin or interphase nuclei to be reprogrammed in the absence of cell division.

Germinal vesicle (GV) stage oocytes do not replicate or divide. They therefore provide an opportunity to test whether the cell cycle phase of donor nuclei affects the efficiency of nuclear reprogramming as judged by active transcription of previously silenced genes [12]. To our surprise, we found that a mitotic state of donor nuclei dramatically increases the efficiency of activating certain quiescent pluripotency genes in these nuclei. Our results support an idea that a brief period during mitosis facilitates an exchange of gene regulatory factors on chromatin and that this could be an important mechanism to help cells embarking on new cell lineages during normal development.

## Results

### Mitotic Chromatin Is Reprogrammed Much More Rapidly Than Interphase Nuclei

**Figure 1:**
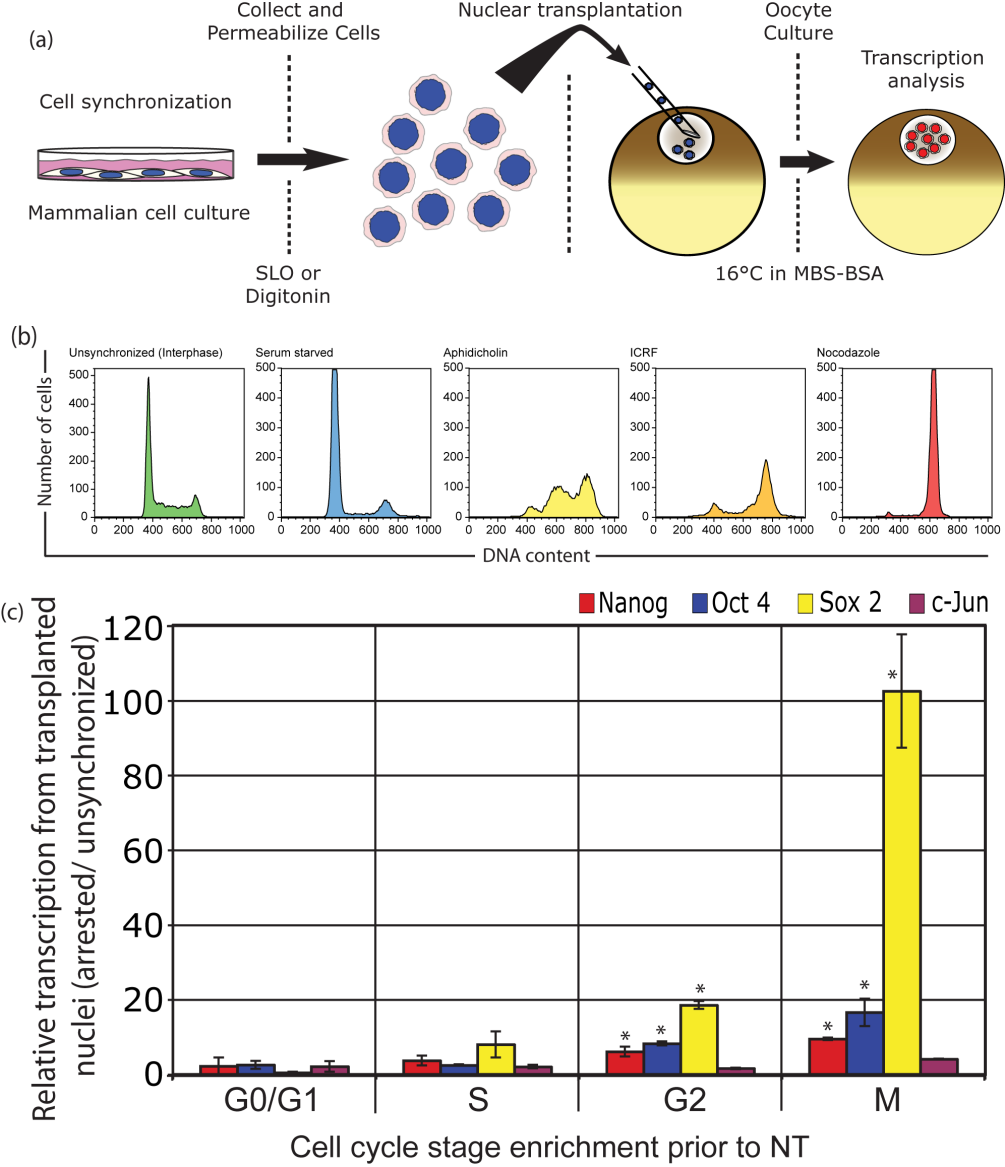
Mitotic nuclei are reprogrammed much more efficiently than interphase nuclei. (a) Nuclear transplantation procedure used in this and the following experiments. (b) DNA content analysis of donor cells used for nuclear transplantation to oocytes confirms enrichment of specific cell cycles stages. (c) Donor nuclei in the later stages of the cell cycle reprogram better than those from earlier stages. Nuclei from C2C12 cells arrested at each stage of the cell cycle or growing in the absence of inhibitor were used as donor material for NT to oocyte GVs. The figure shows the relative expression for each of the indicated genes at 38 h after transplantation compared to unarrested donor cells (*n* = 3). Supporting data can be found in Data S1.

Permeabilized mouse C2C12 cells, a cultured myoblast cell line which we have used extensively in our oocyte nuclear transfer experiments, were arrested at specific stages of the cell cycle (Figure S1a) and were injected into the GV of oocytes (Figure 1a). The DNA content of these donor cell populations (Figure 1b) confirmed cell cycle arrest in each of the cell cycle stages. The transcriptional reactivation of three silent genes quiescent in C2C12 cells (Nanog, Oct4, and Sox2) was assessed by RT-qPCR 38 h after nuclear transplantation (Figure 1c). Nuclei at a late stage of the cell cycle (M) show greatly enhanced transcription of each of the genes when compared to unsynchronized nuclei (predominantly G1 and S), whereas an already active gene (c-jun) shows little increase in transcript level. Particularly impressive is the 100-fold enhancement in Sox 2 expression from mitotic donor nuclei when compared to interphase donor nuclei (Figure 1c). In over 50 experiments, donor cells arrested in mitosis or in late G2 always generated more Sox2 transcripts from reactivated genes at 25–48 h after injection to oocytes than unsynchronized donor cells. This difference ranged from a few fold to over 100-fold and is much affected by the exact duration of nocodazole treatment. Sox2 is a gene that is more widely expressed than most others, notably in early embryos, in most stem cells, and in the nervous system [13].

To test whether this result is a peculiarity of this donor cell type (C2C12 myoblasts) or is a nonspecific effect of nocodazole, we repeated these experiments with 10T1/2 donor nuclei (Figure S1b) or prepared mitotic C2C12 donor cells without any inhibitors by a shake-off procedure (Figure S1c). In both cases, enhanced transcription from mitotic donors was observed, although the magnitude of mitotic advantage was lower (particularly in the case of the shake-off samples, many cells of which appeared to be apoptotic by visual inspection). Mitotic donor nuclei were also prepared using another cell synchronization agent (Taxol), and the mitotic advantage was again seen (Figure S1d). When G1/G0 cells were exposed to nocodazole for the same period of time as used to prepare mitotic cells, no enhancement of transcription of the genes was observed (Figure S1e). These results indicate that the observed mitotic advantage is not due to a nonspecific activity of nocodazole nor to a peculiarity of one line of cells (C2C12).

To ask if this mitotic advantage applies more widely in the genome than to the pluripotency genes so far tested, we compared by RNAseq the genes transcribed in injected oocytes by interphase nuclei or mitotic chromatin. We focussed our analysis on genes that were found to be consistently expressed by interphase nuclei after nuclear transfer. One experiment indicated that 617 genes were transcribed in oocytes at least 2-fold more in mitotic nuclei compared to interphase nuclei. Of these mitotically up-regulated genes, Sox2 was 4-fold more transcribed than in interphase nuclear transfers, and over half of the 617 genes were more strongly transcribed than Sox2. The list of these genes is in Table S1.

### Mitotic Advantage Is Due to an Increased Rate of Reprogramming

**Figure 2:**
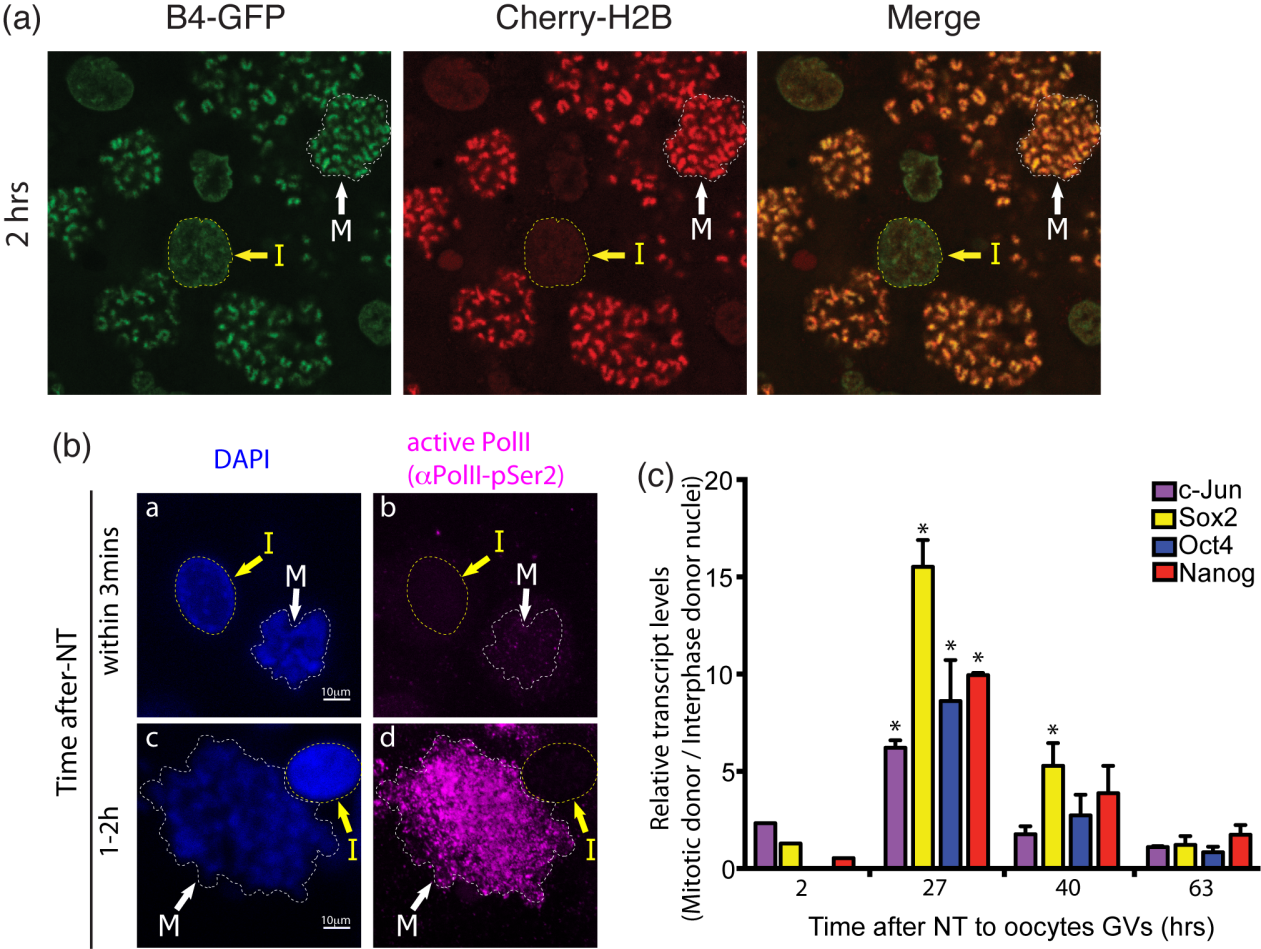
Mitotic advantage is due to an increased rate of reprogramming. (a) Live imaging of mixed mitotic and interphase nuclei injected into the GVs of oocytes expressing fluorescently labelled histone B4 or histone H2B. Mitotic chromatin becomes decorated with oocyte-derived factors to a far greater degree than interphase nuclei 2 h after nuclear transplantation. The arrows indicate interphase (I) or mitotic (M) nuclei. (b) Mitotic donor nuclei (M) display actively transcribing RNA Pol II far more rapidly than interphase nuclei (I) when these nuclei are co-injected into oocyte GVs. Immunofluorescent staining against a mixture of interphase and mitotic donor nuclei injected into the oocyte GV and fixed at 3 min or between 1 and 2 h after transplantation using antibodies against active poI II (magenta). (c) A time course of expression by mitotic and interphase donor nuclei shows that the difference between these nuclei decreases with time, suggesting that the eventual amount of reprogramming is similar in the two nuclear types but that initiation of transcription is much more rapid in mitotic nuclei. Supporting data can be found in Data S1.

The enhanced reprogramming from mitotic donor material could be due to an increased rate or to a greater eventual level of reprogramming. To distinguish these ideas and to measure the rate of reprogramming, we measured the incorporation of GFP-tagged histone B4 (an early marker of oocyte reprogramming) [14] and the association of Cherry-labelled histone H2B by live imaging of mixed populations of mitotic and interphase donor cells after injection into oocytes (see Figure S2a for design). Mitotic donor material becomes very rapidly marked with both histone B4 and histone H2B, whereas interphase donor nuclei show a lag in the association of both and particularly of H2B (Figure 2a). In support of a difference in the rate of reprogramming, we find that oocyte-derived TBP2 marks the transplanted mitotic cells more strongly than interphase donor cells (Figure S2b; compare white mitotic with yellow interphase arrows). We then asked if there is a more rapid association and activation of RNA polymerase II with mitotic chromatin. We used immunostaining for the elongating form of RNA polymerase II on a mixed population of mitotic and interphase nuclei injected into oocyte GVs. Mitotic donor material is clearly marked with elongating Pol II before interphase donor material (Figure 2b, compare panels ii and iv for pol II). In view of this difference between the two nuclear types in the onset of global pol II transcription after nuclear transfer, we asked whether reprogrammed genes are activated at a different rate in mitotic donor cells compared to interphase cells or if the magnitude of activation is greater. A time course of reprogramming from oocytes injected with either interphase or mitotic donor cells was assessed by RT-qPCR and revealed that genes from mitotic donor cells are activated more rapidly than the same genes from interphase cells (Figure 2c); the accumulation of transcripts reached by 63 h is similar.

We conclude that the difference in reprogramming between interphase and mitotic donor material giving this mitotic advantage reflects the rate of reprogramming rather than the eventual magnitude of transcript generation from these two types of nucleus.

### Mitotic Advantage Is Independent of Nuclear Membrane Permeability

**Figure 3:**
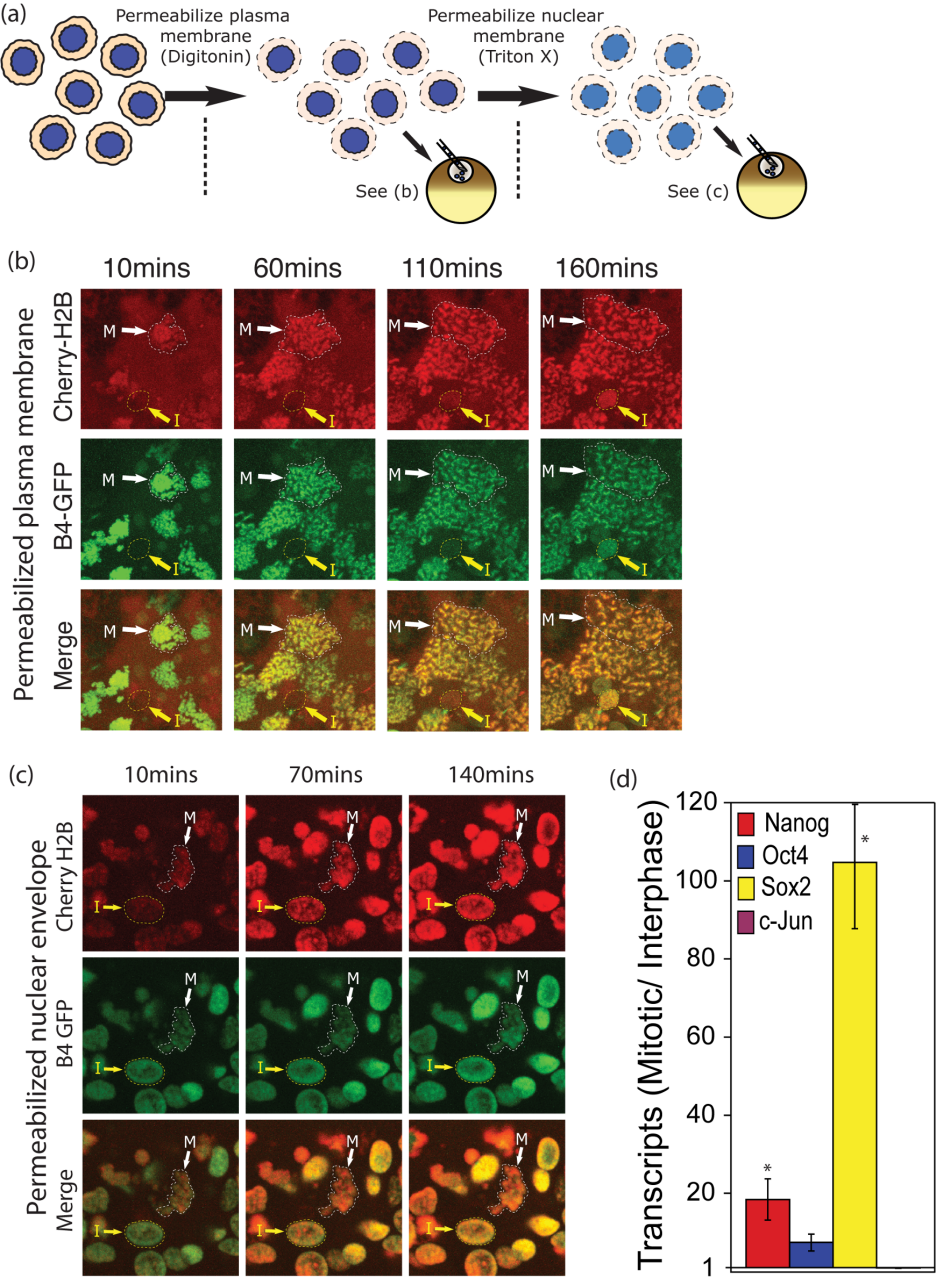
Mitotic advantage is independent of nuclear membrane permeability. (a) Design of permeability assay. (b) Under normal conditions of plasma membrane permeabilization by digitonin with no nuclear permeabilization, mitotic chromatin (M arrows) takes up histones B4 and H2B faster than interphase nuclei (I arrows). (c) When double permeabilized by Digitonin and Triton X, interphase nuclei and mitotic chromatin take up these histones at a similar rate. (d) After double permeabilization, the mitotic advantage of mitotic nuclei is still very large, as judged by RT-qPCR. Supporting data can be found in Data S1.

The most obvious explanation for this mitotic advantage is the absence of a nuclear envelope in the mitotic karyoplasts. We have quantitated this difference in membrane permeability by time course imaging a mixture of injected interphase nuclei and mitotic karyoplasts. We carried out a “double permeabilization,” in which both the cell and nuclear membranes, of interphase or mitotic donor cells, were permeabilized as illustrated in the scheme in Figure 3a. We then compared the rate of oocyte factor uptake with the rate of reprogramming by RT-qPCR. A difference in the amount of B4 and H2B uptake is indeed seen after plasma permeabilization with digitonin (Figure 3b) but is no longer seen after double permeabilization of the nuclear envelope with Triton (Figure 3c). Nevertheless, the mitotic difference between interphase and mitotic chromatin does persist in respect of the transcriptional reprogramming of silenced genes (Figure 3d).

We have confirmed this conclusion using permeabilization by different reagents. Streptolysin 0 (SLO) permeabilizes the plasma membrane but not the nuclear membrane; SLO and Lysolecithin (LL) together permeabilize the plasma membrane and nuclear membrane [15]. Permeabilization was tested using different sizes of dextran (Figure S3a). We then compared transcription from transplanted nuclei, comparing those treated with SLO alone and those treated with SLO and LL. The transcription ratio following these two procedures shows no advantage when the nuclear envelope is permeabilized (Figure S3b).

We conclude that the presence of an intact interphase nuclear envelope does not explain the mitotic advantage.

### The Mitotic Advantage Is Due to Chromatin Composition

**Figure 4:**
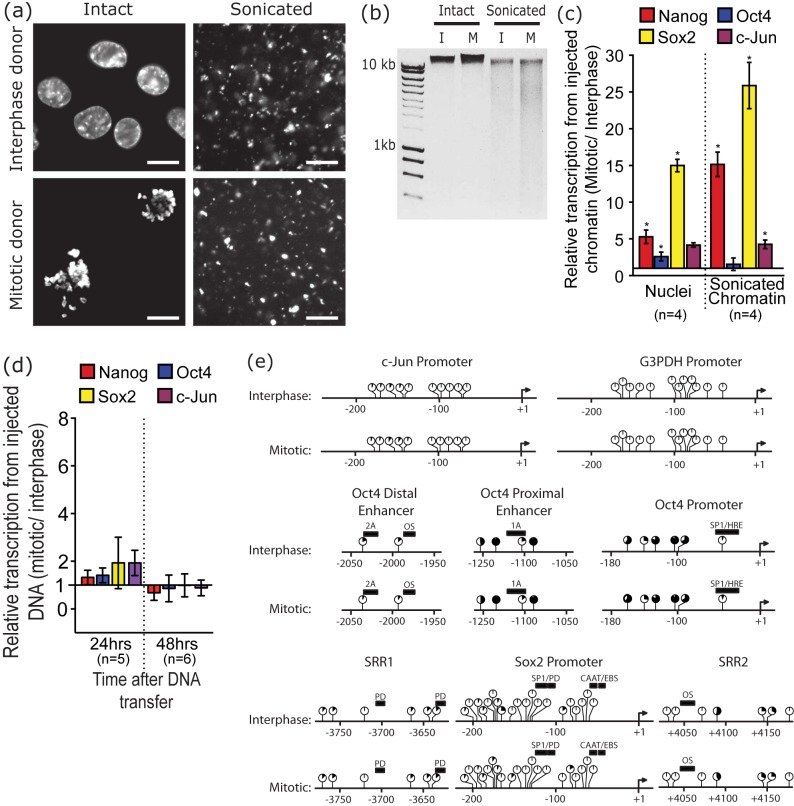
Sonication does not eliminate mitotic advantage. (a) Interphase and mitotic donor nuclei were mildly sonicated to fragment the chromatin as shown by DAPI staining of the four kinds of donor nuclei. (b) The major proportion of DNA in both sonicated samples is above the size exclusion limit of the gel, confirming mild sonication. (c) Interphase and mitotic nuclei or corresponding sonicated chromatin preparations were transplanted into oocyte GVs and gene reactivation analyzed by RT-qPCR after 42 h. The mitotic advantage is retained on fragments of chromatin. Supporting data can be found in Data S1. (d) Genomic DNA prepared from interphase and mitotic cells was injected into oocyte GVs and gene transcription assessed by RT-qPCR. There is no significant difference between interphase and mitotic DNA with respect to gene activation in the oocyte at either of the indicated time points. Supporting data can be found in Data S1. (e) There is no observable difference in DNA methylation between interphase and mitotic cells as determined by pyrosequencing of bisulphite-converted genomic DNA (horizontal lines represent the indicated DNA sequences, with balls representing individual CpG dinucleotides; black filling represents the percentage of methylation for each site). Solid black bars represent the positions of known transcription factor binding sites, such as SP1/HRE. OS is Oct-Sox, PD is Pou-Domain, and SRR is the Sox2 Regulatory Region, and genomic distances are presented below each map, set relative to the transcriptional start site of each gene.

Because the difference in reprogramming rate between interphase and mitotic donor cells is maintained after extensive permeabilization of the interphase nuclear membrane, we asked if the source of the difference lies in the chromatin of the two donor cell preparations. To answer this, we mildly sonicated both interphase and mitotic donor cell preparations to give fragments of chromatin of similar sizes (Figure 4a and b), injected these preparations in parallel with a permeabilized cell preparation into oocyte GVs, and assessed gene reactivation by RT-qPCR (Figure 4c). It is clear that the difference in the rate of gene reactivation from interphase and mitotic nuclei is maintained when the injected material is sonicated chromatin as opposed to whole nuclei. This suggests that the “mitotic advantage” is present in the chromatin of mitotic cells. This result also confirms that the difference between interphase and mitotic donor cells is not due to the interphase nuclear membrane, nor to any other aspect of nuclear organization that is eliminated by sonication.

The difference between interphase and mitotic reprogramming is, however, abolished when genomic DNA prepared from donor nuclei is injected into oocyte GVs (Figure 4d); this excludes differences at the DNA level (sequence and DNA methylation for example) as possible sources of the difference in reprogramming between interphase and mitotic samples. The possibility of DNA methylation accounting for the mitotic effect was further excluded by bisulphite analysis of specific loci on mitotic and interphase DNAs, as this revealed no mitosis-specific differences (Figure 4e). These two results indicate that whatever accounts for the difference between mitotic and interphase donor cells is not present at the level of genomic DNA itself but is in non-DNA components of chromatin.

### Mitotic Advantage Is Independent of Most Salt-Released Chromatin Factors

**Figure 5:**
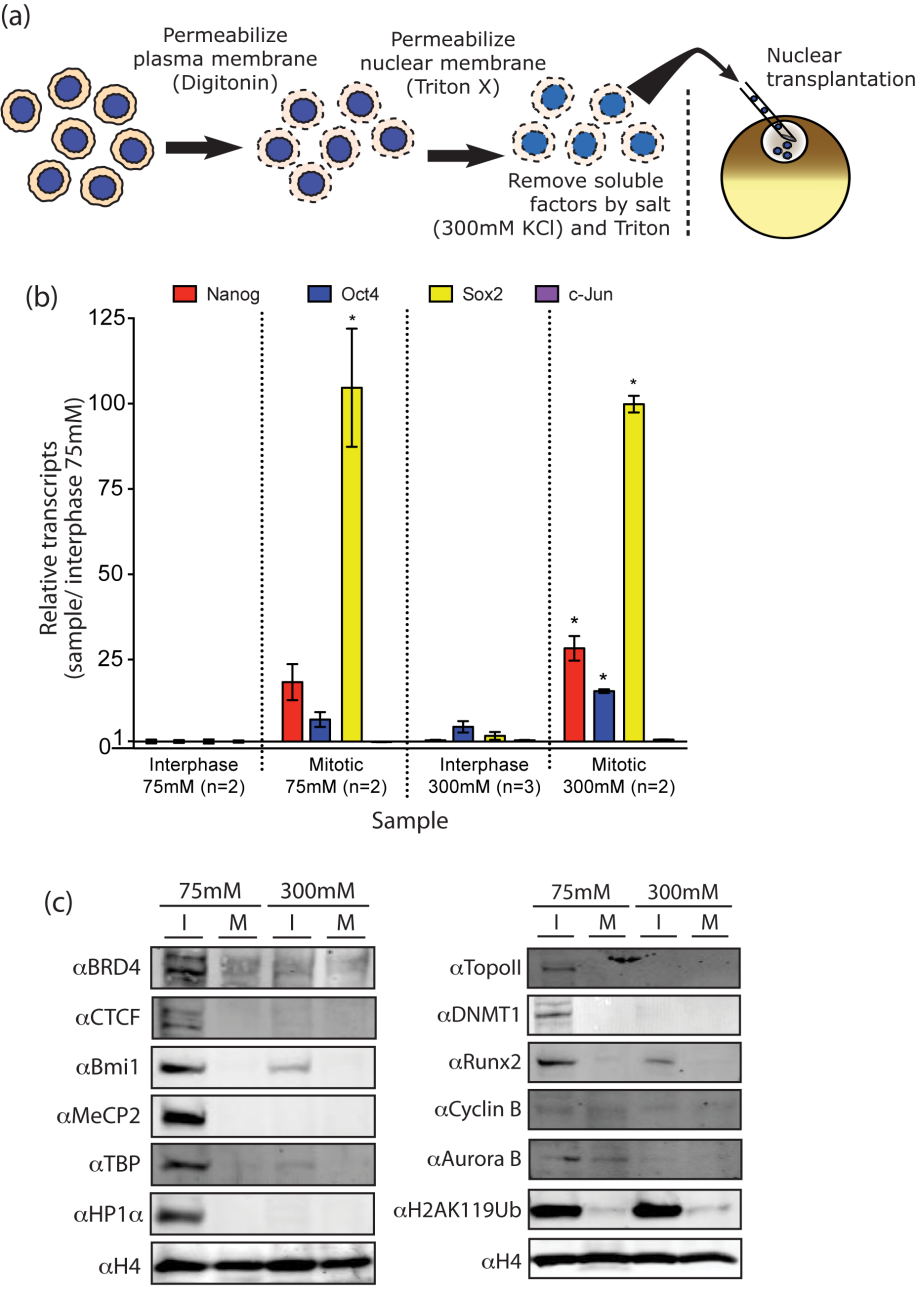
Removal of salt-soluble factors from interphase C2C12 nuclei. (a) Design of salt depletion procedure. (b) 300 mM salt does not remove the mitotic advantage of mitotic nuclei, nor does it make interphase nuclei behave like mitotic chromatin by loss of DNA-binding factors. Salt-treated samples were injected to oocytes and cultured for 40 h and then analyzed by RT-qPCR. Supporting data can be found in Data S1. (c) Two independent experiments show that the majority of chromatin binding factors can be depleted from nuclei by 300 mM salt and Triton when compared to 75 mM salt, which should not remove chromatin-bound factors. The blemish for topoisomerase II (75 mM,M) is not in the position of this protein.

As the mitotic advantage is likely to be due either to the loss or gain of chromatin binding factors, we removed most of these from our donor suspension of interphase nuclei by incubating such nuclei in a high-salt Triton buffer. We thereby tested whether a loss of chromatin binding factors at mitotic entry could remove the mitotic advantage. We also largely depleted chromatin binding factors from permeabilized mitotic cells and thus removed many chromatin factors that may be gained by cells entering mitosis. The depletion of chromatin binding factors was achieved with 300 mM salt and Triton, which removed most nonhistone DNA binding factors. A scheme of the cell preparation and examples of the proteins removed are shown in Figure 5a and 5c. It can be seen that the great majority of the nonhistone chromosomal proteins that have been tested and that normally exist in interphase nuclei have been removed from mitotic chromatin by 300 mM salt and Triton. Nevertheless interphase nuclei depleted of salt soluble nuclear protein (300 mM sample) do not acquire the same reprogramming responsiveness as mitotic donor material (Figure 5b). Likewise, extensive protein removal from mitotic donor material (Figure 5c) before nuclear transplantation does not abolish the mitotic advantage (mitotic 300 mM; Figure 5b), indicating that the acquisition of chromosomal proteins by mitotic nuclei does not account for this advantage.

Independently of salt release experiments, we tested topoisomerase II whose activity increases from S phase to the end of G2. The inhibition of topoisomerase II and of its adaptor molecules 14-3-3z and H3S10ph by inhibitors, inhibitory peptides, and antibody injection in transplanted mitotic nuclei did not reduce the mitotic advantage. We also found that salt release removes topoisomerase from mitotic chromatin, as in experiments shown in Figure 5c, but does not change the mitotic advantage.

We conclude that a loss of salt-soluble chromatin binding factors does not account for the mitotic advantage. It is likely therefore that the source(s) of the difference is either a non-salt-soluble factor (gained or lost at mitotic entry), a covalent modification of chromatin, or the spatial arrangement of nucleosomes.

### Histone Acetylation, Phosphorylation, and Methylation

**Figure 6:**
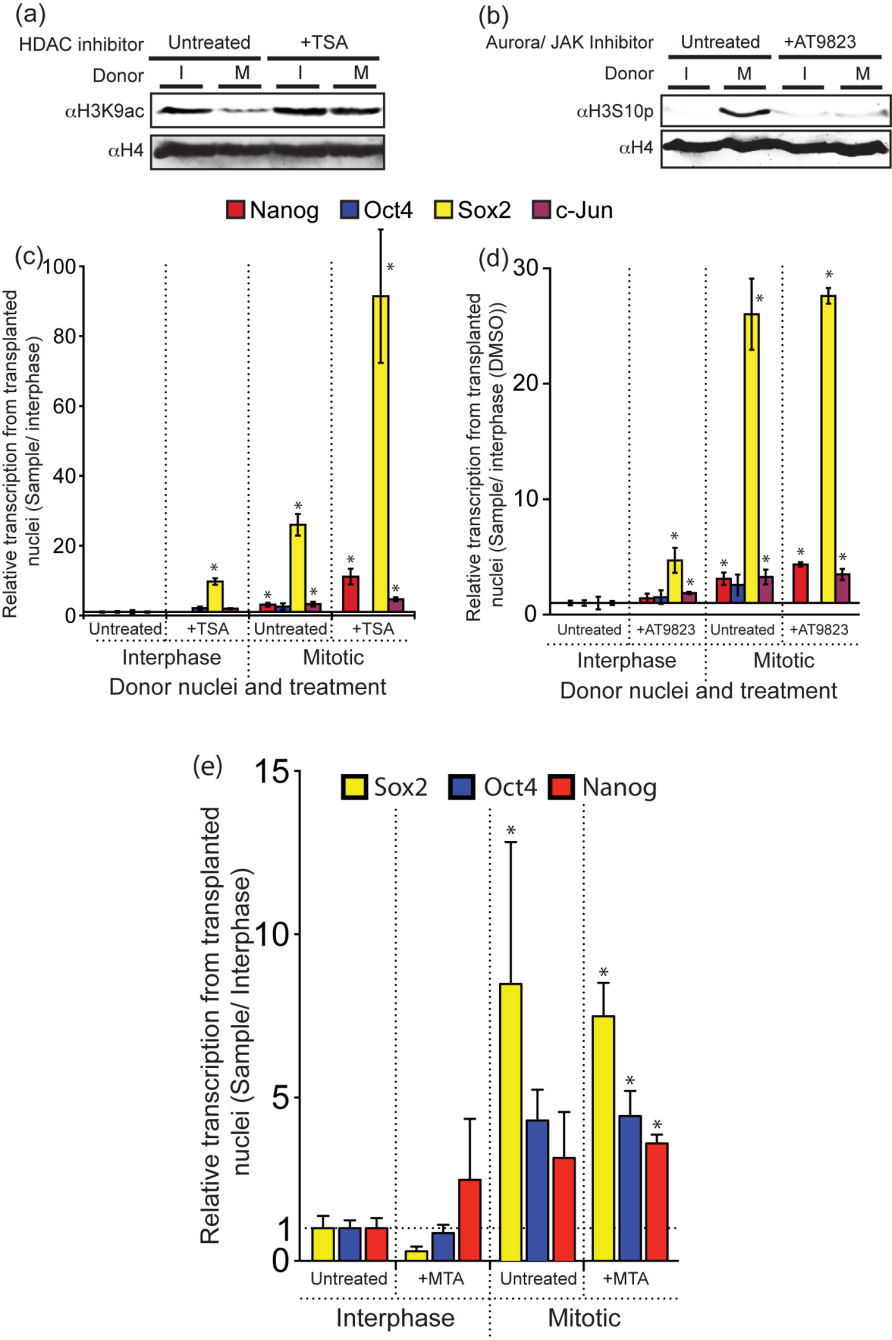
Most mitotic histone modifications do not distinguish mitotic chromatin from interphase nuclei. (a) Western blot showing TSA inhibition of the normal mitotic deacetylation of histone H3K9ac. (b) Western blot showing that AT2983 prevents the normal mitotic phosphorylation of histone H3. (c) Mitotic nuclei prepared in the presence of TSA maintain the mitotic advantage. Supporting data can be found in Data S1. (d) Inhibiting normal mitotic phosphorylation by AT2983 has no adverse effect on mitotic advantage. Supporting data can be found in Data S1. (e) MTA inhibits mitotic methylation of H3K4me3 and H3K9me3, both of which are normally increased at the Sox2 locus in C2C12 cells. This does not change the mitotic advantage. Supporting data can be found in Data S1.

We next considered covalent histone modifications that may be lost or gained on mitotic chromatin compared to interphase chromatin. A large number of histone modifications are associated with mitotic entry [16],[17], as well as changes in nucleosome positioning and in chromatin compaction. We first tested the most striking changes involving global histone deacetylation, phosphorylation, and some small increases in histone H3 lysine 4 and 9 methylation that have been seen on mitotic chromatin [16],[18],[19]. Histone deacetylation in mitotic cells is successfully inhibited during mitotic synchronization by the histone deacetylase inhibitor TSA (Figure 6a). Histone phosphorylation in mitotic cells is inhibited by the Aurora B/JAK inhibitor AT9823 (Figure 6b). Nevertheless, the mitotic advantage persists after both of these treatments (Figure 6c and 6d). Similarly, the removal of mitotic histone phosphorylation from the Sox2 gene by protein phosphatase treatment of mitotic and interphase donor cells before nuclear transplantation also failed to abrogate the mitotic effect (Figure S4a and b). A small (2-fold) local increase in Sox2 locus histone methylation at mitosis (Figure S4c and d), seen by ChIP in mitotic chromatin, is eliminated by the methylation of MTA (not shown), but the mitotic advantage is retained (Figure 6e).

### Histone Ubiquitination

**Figure 7:**
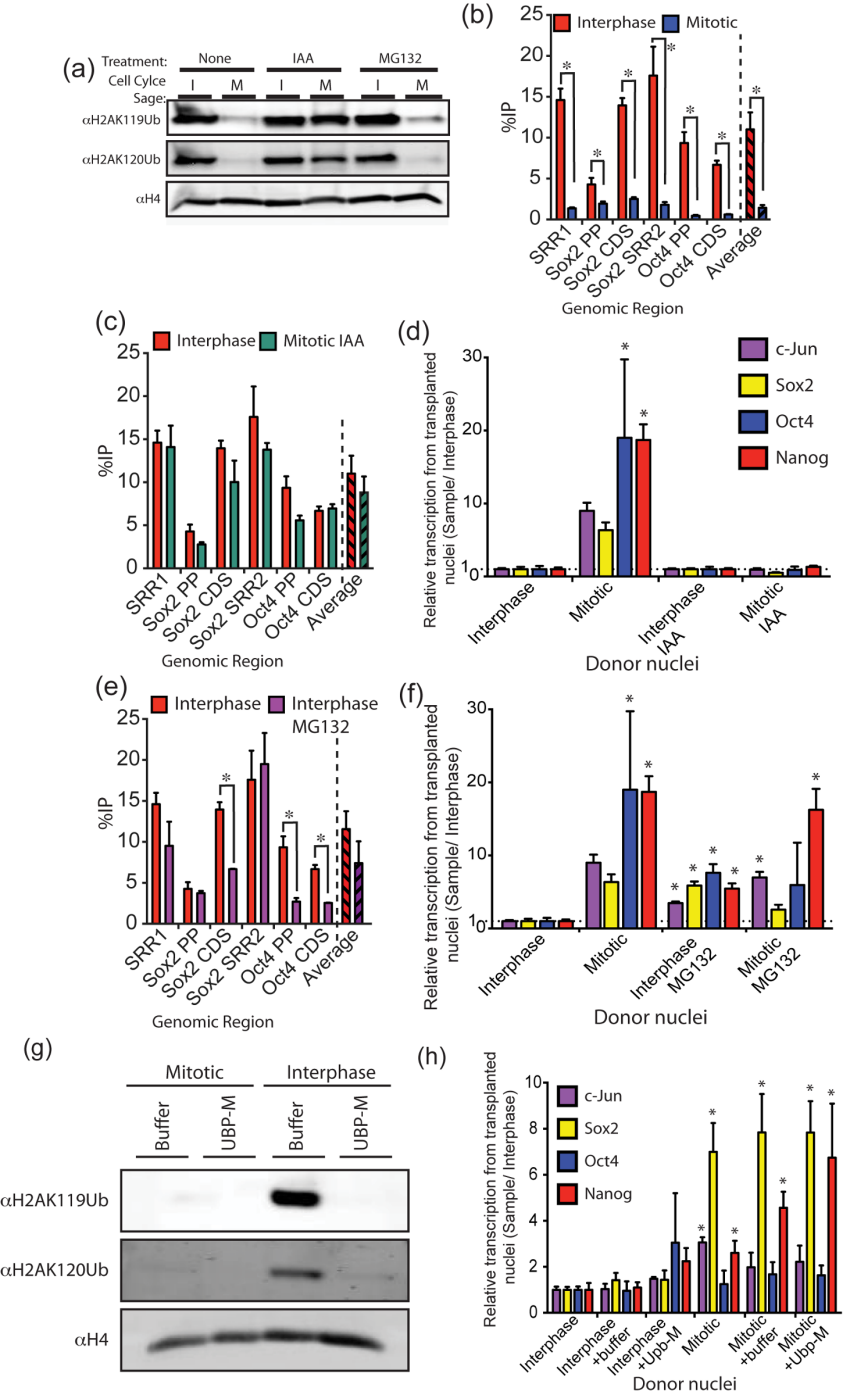
Histone ubiquitination can explain the mitotic advantage. (a) Western blot for histone H2AK119Ub and H2B120Ub from interphase and mitotic donor cells treated with IAA or MG132. (b) ChIP analysis for ubiquitinated histone H2A shows a large difference between mitotic chromatin and interphase nuclei for several genomic regions. (c) IAA largely removes the deubiquitinated state of mitotic chromatin in several gene regions. None of these values are significantly different from one another. Supporting data can be found in Data S1. (d) Mitotic advantage is eliminated by IAA treatment. Supporting data can be found in Data S1. (e) H2AK119 ubiquitination in interphase nuclei is reduced by MG132. Supporting data can be found in Data S1. (f) RT-qPCR of interphase and mitotic nuclei treated with MG132 after nuclear transfer to oocytes; interphase transcription is much enhanced. Supporting data can be found in Data S1. (g) Western blot to show *in* vitro deubiquitination of interphase and mitotic donor nuclei. (h) RT-qPCR transcription analysis of Ubp-M-treated donor nuclei 36 h after nuclear transfer. Supporting data can be found in Data S1.

In normal cells, histone ubiquitination (primarily H2AK119Ub and H2BK120Ub) is dramatically reduced at mitotic entry [16]. H2AK119Ub is associated with transcriptional repression [20]. Thus, a reduction in H2A ubiquitination at mitosis is an attractive candidate to explain the enhanced reprogramming of mitotic chromatin. We first tested the effect of increasing ubiquitination of mitotic chromatin, by preparing nuclei for injection in the presence of iodoacetimide (IAA), which inhibits deubiquitinases (Figure 7a) [21]. Under normal conditions, interphase chromatin is at least five times more globally ubiquitinated than mitotic chromatin (Figure 7a,b). The inhibition of mitotic deubiquitination by IAA increases the ubiquitin level in mitotic chromatin, so that it is nearly equal to that of interphase nuclei (Figure 7c). When tested for transcription in oocytes, hyperubiquitinated mitotic chromatin by IAA does not show an advantage over interphase chromatin (Figure 7d), in accord with the idea that the deubiquitinated state of normal mitotic chromatin could account for its special transcriptional advantage.

As further support for this idea, we tried to remove ubiquitin from interphase nuclei with a recombinant deubiquitinase (Ubp-M), and then tested the effect of this by oocyte injection followed by RT-qPCR. Treatment of interphase nuclei with Ubp-M removes histone ubiquitination (Figure 7g). However, unexpectedly, we see that the removal of histone ubiquitination by Ubp-M does not significantly enhance the reprogramming of interphase nuclei, so that they behave the same, in this respect, as mitotic chromatin (Figure 7h). This suggested that deubiquitination itself is not sufficient to confer mitotic advantage. We hypothesized that H2A deubiquitination is a required step in a series of chromatin remodelling events that eventually lead to mitotic advantage. We therefore chose to reduce the ubiquitinated state of interphase nuclei in living cells in order to allow events downstream of ubiquitin-depleted chromatin to take place. The inhibitor MG-132 is thought to lower histone ubiquitination by reducing the pool of free ubiquitin through inhibition of the proteasome. MG-132 treatment of interphase nuclei before injection to oocytes (Figure 7a) gave a partial but significant reduction in H2A ubiquitination on Sox2 (Figure 7e compared to 7b); it also resulted in a substantial enhancement of oocyte-induced transcription from interphase, but not mitotic chromatin (Figure 7f).

The increase in ubiquitination of transplanted mitotic nuclei, coupled with the removal of the mitotic advantage, shows that chromatin ubiquitination contributes to the mitotic advantage. It may, however, not be sufficient to explain the whole phenomenon, because we do not achieve a complete mitotic advantage in interphase nuclei by deubiquitination.

## Discussion

Our results show a substantial effect of the cell cycle stage of donor nuclei in nuclear transfer experiments. The reason why this has not been seen before in some of the older nuclear transfer experiments with eggs is probably for several reasons. One is that the somatic nuclei used as donors were not able to be well synchronized [22]–[24]. Another is that tests have involved the normality of development, rather than gene activity. Third, and most importantly, tests have been carried out on cell dividing eggs, whereas our work has tested gene transcription in the complete absence of DNA replication or cell division. The more recent results of [6] are in agreement with the work described here. The success of the first mammal cloning work was attributed in part to the use of donor cells in G0 [25],[26]; this result was not, however, found by others [27]–[30]. Lemaitre et al. [6] have described a dramatic effect of mitosis on the efficiency of DNA replication by egg cytoplasm, but transcription could not be tested in their extract experiments. Egli et al. [3] have proposed an important role for mitosis in permitting chromosomal protein exchange in mouse nuclear transfer experiments but not necessarily on gene transcription. Our results are therefore in accord with previous work, but reveal a specific effect of mitosis on gene transcription.

An important component responsible for the acquisition of mitotic advantage appears to be the removal of ubiquitin from histone H2A or H2B in mitotic chromatin. Ubiquitination of histones is primarily monoubiquitination, and we assume that this is the modification involved in mitotic advantage. H2A ubiquitination on lysine 119 is associated with transcriptional repression, particularly of lineage-specifying genes in ES cells [31], possibly through its association with members of the polycomb repressive proteins [32]. It could be envisaged that the deubiquitination of chromatin seen at mitotic entry permits this mitotic advantage by removing this inhibitory mark or the associated binding proteins [33]. In keeping with this idea, we have been able to partly simulate the mitotic effect by biochemically removing histone ubiquitination in interphase donor cells.

What could be the significance of the mitotic advantage identified here? In our experiments, the mitotic advantage takes place during the early stages of transcriptional activation, and is no longer seen after 2 d. In normal dividing cells, mitosis lasts for only a few hours. We therefore think that mitosis is a time when cells can most easily change their chromatin state, exchange transcription factors, and embark on a new lineage. When a cell has adopted a new fate, its daughter cells will usually follow the same lineage, unless an exchange of nuclear components takes place. The acceleration of postmitotic transcriptional activation [34] may be an associated phenomenon.

## Materials and Methods

### Cell Culture

Cells were cultured in DMEM (D5796, Sigma; E15-810, PAA; 41965-062, Invitrogen) with 10% FCS (10270106, Invitrogen), 100 units/ml Penicillin-Streptomycin (15140-122, Invitrogen), and 0.25 µg/ml Fungizone (15240-096, Invitrogen). Inhibitors used for various experiments include the following: 5′-Deoxy-5′-(methylthio)adenosine (MTA)(D5011, Sigma) used at 1 µg/ml, Aphidicolin (A0781, Sigma) at 1 µg/ml, AT9823 (gift from M. Dawson) used at 100 nM; ICRF-90 (I4659, Sigma) at 1 µg/ml, iodoacetamide (IAA) made freshly and used at 10 µM, MG-132 (Sigma) used at 4 µM, Nocodazole (M1404, Sigma) at 75–100 nM; Taxol (T7402, Sigma) at 1 µM, Thymidine (T1895, Sigma), and Trichostatin A (T8552, Sigma) at 1 µg/ml.

### Synchronization

Cell synchronization was achieved according to the scheme in Figure S1A. In general, media containing the desired inhibitor were applied to unsynchronized cells a day after seeding for 16–20 h. For mitotic cells, seeded cells were initially arrested in 2 mM thymidine for 16–24 h, washed 3× in PBS, released into fresh media for 6–12 h, and then media replaced with Nocodazole or Taxol containing media for 10–16 h, after which rounded cells were detached by “shake-off” and the culture media harvested for the mitotic cell fraction. G1 arrest was achieved by Serum starvation for 72 h.

### Nuclear and Karyoplast Preparation and Transplantation to Oocyte GVs

Cells in suspension (either from mitotic shake-off or trypsinization of adherent cells) were washed twice in PBS, transferred to SuNaSP, and permeabilized with Digitonin (40–100 µg/ml) for 3 min on ice. The reaction was stopped by addition of and excess of SuNaSP-BSA and the nuclei concentrated to an appropriate volume for GV transfer [12]. Nuclear transplantation to oocyte GVs was performed as described in [12].

### Media for Permeabilization (Nuclear Suspension Media)

The following media were used for permeabilization: SuNaSP, 0.25 M Sucrose, 75 mM NaCl, 0.5 mM Spermidine, 0.15 mM Spermine; SuNaSP-BSA, SuNaSP with 3% (w/v) BSA; and HPRicLS, Final [1×] Hepes 20 mM, KCl 75 mM, MgCl_2_ 1.5 mM.

### Salt Depletion of Donor Cells

Cells were permeabilized with Digitonin and incubated for 15 min in prebuffer (20 mM Hepes, 75 mM KCl, 1.5 mM MgCl_2_, 25 mg/ml Gelatin, 60 mg/ml BSA) and washed twice into permeabilization buffer (20 mM Hepes, 75 or 300 mM KCl, 1.5 mM MgCl_2_, 0.2% TritonX100, 12 mg/ml Gelatin, 30 mg/ml BSA). Cells were then extensively washed (20 mM Hepes, 75 mM KCl, 1.5 mM MgCl_2_) and resuspended in a suitable volume of SuNaSP-BSA for nuclear transplantation.

### In Vitro Deubiquitination and Dephosphorylation of Donor Cells

Cells were permeabilized in Digitonin, incubated in “prebuffer,” washed into permeabilization buffer (75 mM salt), washed in SuNaSP, and then transferred into a suitable reaction buffer with or without recombinant enzyme. Dephosphorylation was performed with Protein Phosphatase I (NEB, P0754S) in HPRicLS (20 mM Hepes, 75 mM KCl, 1.5 mM MgCl_2_) and Deubiquitination performed using recombinant enzyme prepared from insect cells (as described in [33]) in buffer (50 mM NaCl, 50 mM Tri-Cl PH 8.0, 1 mM DTT, 1× Complete EDTA-free Protease inhibitor [Roche]). After enzyme treatment, cells were washed in HPRicLS and SuNaSP and resuspended in a suitable volume of SuNaSP-BSA.

### Flow Cytometry

PBS washed cell suspensions were (fixed in ethanol and stained with 50 µg/ml propidium iodide). DNA content analyses were then performed on a FACSCalibur cytometer (BD Bioscience).

### Antibodies for ChIP and Western Blot

The following antibodies were used: αAurura B (AIM1) (Cell Signalling, 3094), αBmi1 (Cell Signalling, 6964), αBRD4 (Cell Signalling, 12183), αCTCF (Cell Signalling, 3418), αCyclinB (Cell Signalling, 4138), αDNMT1 (Abcam, ab92453), αH2AK119Ub (Cell Signalling, 8240), αH2BK120Ub (Cell Signalling, 5546), αH3 (Abcam, ab1791 and Cell Signalling, 4620), αH3K4me3 (Abcam, ab8580), αH3K9ac (Cell Signalling, 9649), αH3K9me2/3 (Cell Signalling, 5327), αH3S10ph (Sigma, H 0412), αH4 (Abcam, ab31830), αHP1α (Cell Signaling, 2616), αphosphoSer2-PolII (Covance, MMS-129R), αRunx2 (Cell Signalling, 8486), and αTBP (Abcam, ab62125).

### RT-qPCR

RT-qPCR was performed as described in [11]. Unless otherwise stated, results are normalized to VegT (correcting for intrasample RNA extraction variation) and G3PDH (correcting for nuclear number differences between injected oocyte samples). Error bars indicate SME or standard deviation, and significance is determined by unpaired Student *t* test, with *p*<0.05 being considered significant. All experiments presented were single experiments representative of at least three experimental repeats unless otherwise noted.

### Bisulphite and Pyro Sequencing

Genomic DNA was prepared using DNeasy blood and tissue kits (69504, Qiagen), bisulphite conversion was performed using EpiTect Bisulfite Kit (59104, Qiagen), and primer sequences for DNA preparation were designed using Qiagen. Pyrosequencing was performed on a Qiagen Pyromark Q96 ID using PyroMark Gold Q96 Reagents (972804, Qiagen) and PyroMark PCR Kit (978705, Qiagen), as per the manufacturer's recommendations.

## Supporting Information

Data S1 Supporting histogram data.
(XLSX)

Figure S1 (a) Design of cell cycle synchronization procedure. (b) 10T1/2 cells show a mitotic advantage by transcription assay after injection into oocytes. Supporting data can be found in Data S1. (c) Shake-off procedure to enrich for mitotic cells without inhibitors also displays some mitotic advantage when compared to interphase cells. The proportion of cells in mitosis for these samples is indicated in the adjacent table. Supporting data can be found in Data S1. (d) Taxol-synchronized cells give the same transcriptional enrichment as nocodazole-arrested cells after transplantation to oocyte GVs. Supporting data can be found in Data S1. (e) G1/G0-arrested cells, treated with Nocodazole, show no enhancement of pluripotency gene transcription after nuclear transplantation to oocyte GVs. Supporting data can be found in Data S1.
(TIF)

Figure S2 Design of experiments for nuclear incorporation assays. (a) A mixture of mitotic and interphase donor nuclei are injected into oocyte GVs, 24 h after the oocytes have been injected with RNAs encoding fluorescently labelled (GFP or Cherry) proteins. The resulting GVs are then isolated from the oocyte and the transplanted nuclei examined by confocal microscopy. I, interphase; M, mitotic. (b) The oocyte-specific transcription factor TBP2 (red) is taken up by mitotic nuclei to a far greater extent than interphase nuclei by 48 h. GFP labelled histone B4 is used to mark the position of transplanted mitotic and interphase nuclei. The arrows indicate examples of one interphase (yellow) and one mitotic nucleus (white).
(TIF)

Figure S3 Permeabilization of the nuclear membrane does not reduce mitotic advantage. (a) 3 KDa dextran enters SLO-permeabilized nuclei, but 70 KDa dextran does not (black spheres). SLO and LL together permit entry of 70 KDa dextran nucleoplasm. The table illustrates the proportion of permeabilized plasma and nuclear membranes by these treatments. (b) Transcriptional reprogramming 24 h after transplantation of SLO or SLO+LL permeabilized nuclei to oocyte GVs is similar. Supporting data can be found in Data S1.
(TIF)

Figure S4 Phosphatase treatment does not decrease mitotic advantage. (a) Phosphatase removes phosphorylation of H3S10 from mitotic nuclei. (b) Phosphatase treatment does not eliminate mitotic advantage. Supporting data can be found in Data S1. (c) MTA may be used to reduce the local methylation of some genes on mitotic entry. ChIP against the Sox2 promoter and coding sequence using an antibody against H2K4me3 and (d) H3K9me2/3. Supporting data can be found in Data S1.
(TIF)

Table S1 List of genes that are up-regulated in mitotic nuclei transplanted to *Xenopus* oocytes when compared to interphase nuclei transplanted to *Xenopus* oocytes. We have identified by RNA-seq genes that are consistently expressed 48 h after nuclear transfer of interphase transformed mouse embryonic fibroblasts to *Xenopus* oocytes (manuscript under consideration). We used this set of 4,210 genes expressed after nuclear transfer in a comparison with one experiment in which mitotic nuclei transcription following nuclear transfer was also measured by RNA-seq. This led to the identification of 617 genes up-regulated from mitotic nuclei compared to interphase nuclei. The table shows the name of these genes, RPKM from transplanted interphase nuclei (mean of three independent experiment), RPKM from transplanted mitotic nuclei (one experiment), as well as the ratio of mitotic to interphase RPKMs.
(XLSM)

## Abbreviations

GV: germinal vesicle
IAA: iodoacetimide
LL: lysolecithin
RT-PCR: reverse transcriptase polymerase chain reaction
SLO: streptolysin O
TBP: tata box binding protein
TSA: trichostatin A
