# Supplementary figures and images for: Mitosis Gives a Brief Window of Opportunity for a Change in Gene Transcription
Source: PLoS Biol. 2014 Jul 29;12(7):e1001914. doi: 10.1371/journal.pbio.1001914 (PMC4114836; doi:10.1371/journal.pbio.1001914)

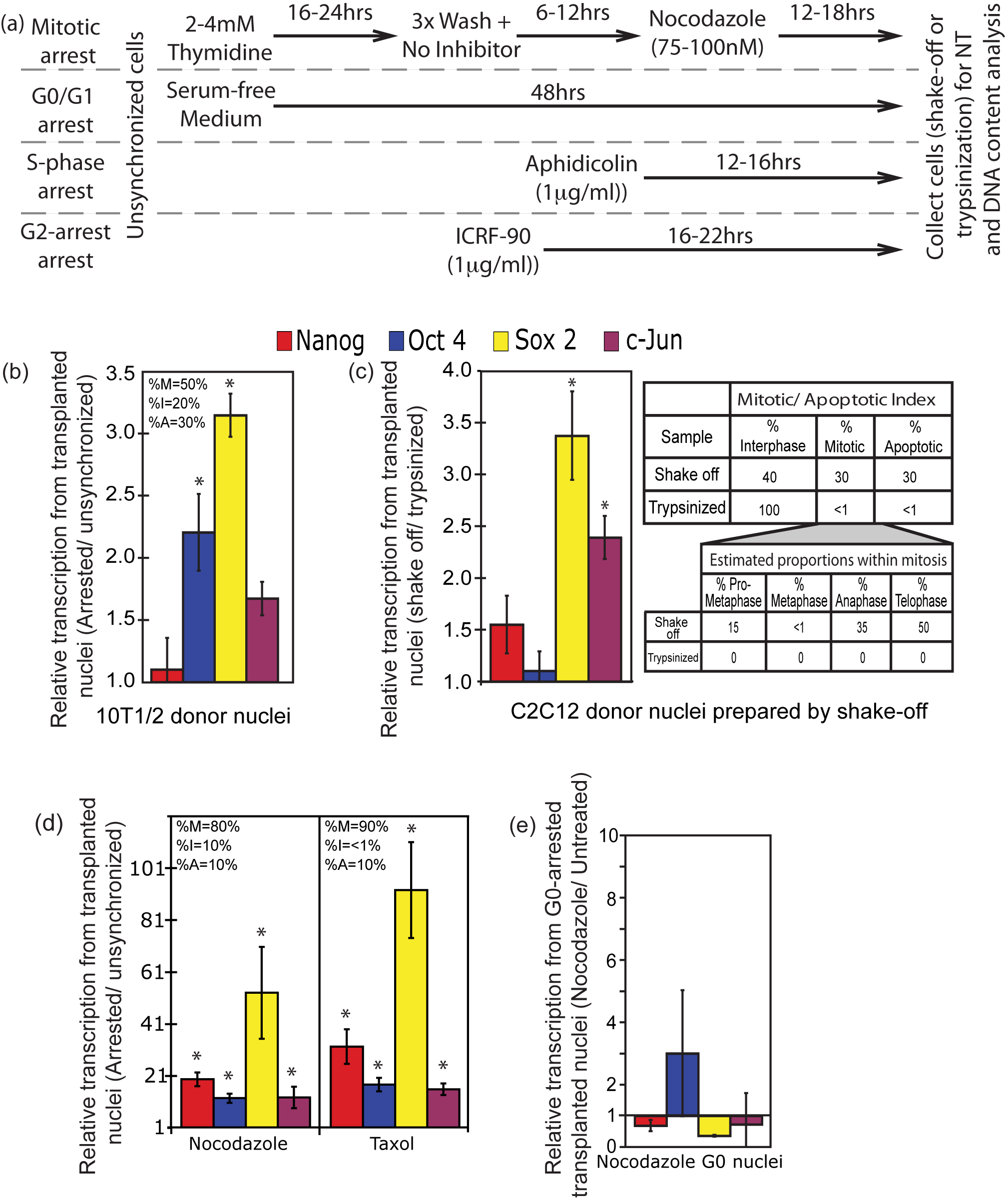

Supplement: Figure S1 — (a) Design of cell cycle synchronization procedure. (b) 10T1/2 cells show a mitotic advantage by transcription assay after injection into oocytes. Supporting data can be found in Data S1. (c) Shake-off procedure to enrich for mitotic cells without inhibitors also displays some mitotic advantage when compared to interphase cells. The proportion of cells in mitosis for these samples is indicated in the adjacent table. Supporting data can be found in Data S1. (d) Taxol-synchronized cells give the same transcriptional enrichment as nocodazole-arrested cells after transplantation to oocyte GVs. Supporting data can be found in Data S1. (e) G1/G0-arrested cells, treated with Nocodazole, show no enhancement of pluripotency gene transcription after nuclear transplantation to oocyte GVs. Supporting data can be found in Data S1. (TIF) [file pbio.1001914.s002.tif]

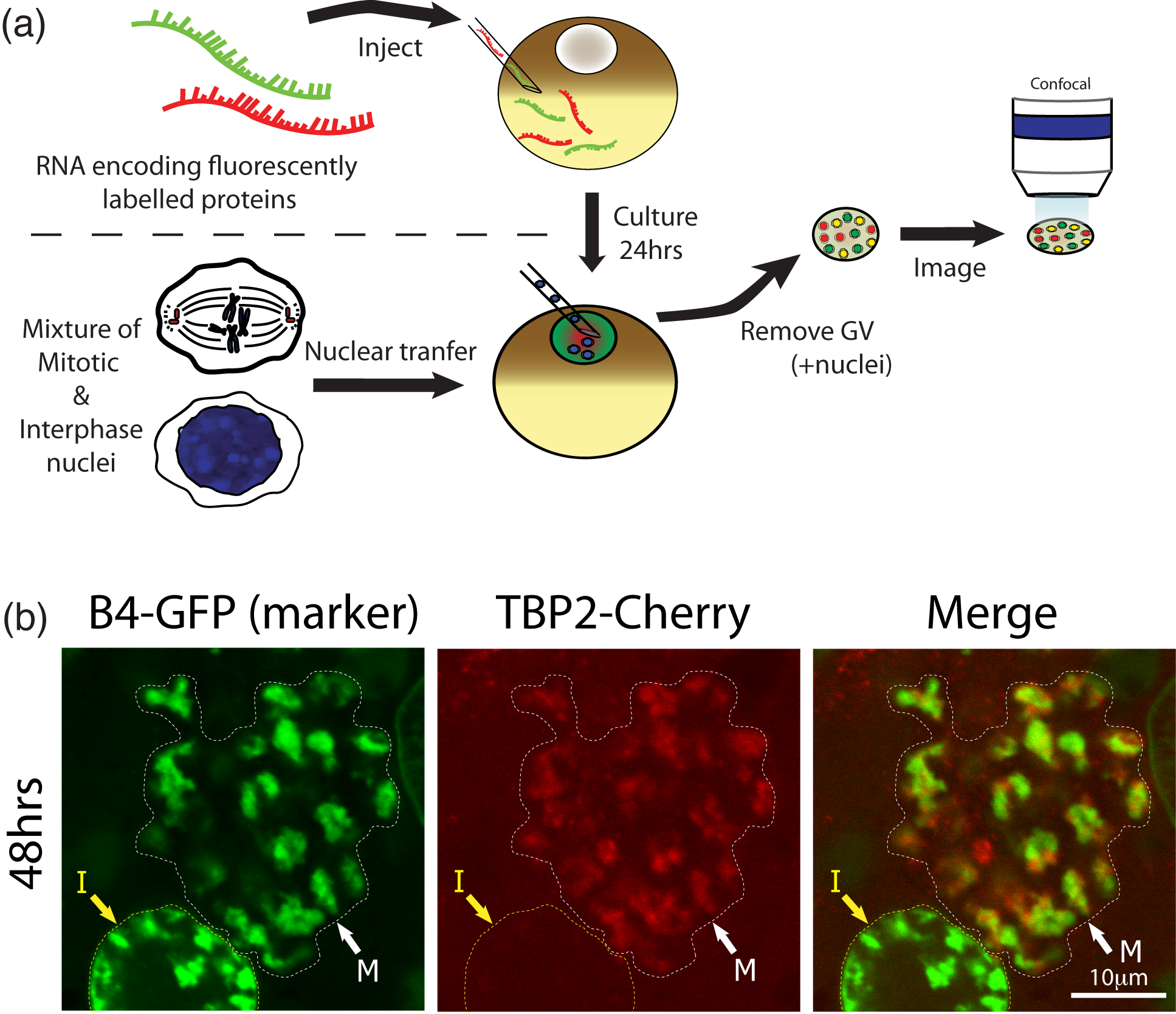

Supplement: Figure S2 — Design of experiments for nuclear incorporation assays. (a) A mixture of mitotic and interphase donor nuclei are injected into oocyte GVs, 24 h after the oocytes have been injected with RNAs encoding fluorescently labelled (GFP or Cherry) proteins. The resulting GVs are then isolated from the oocyte and the transplanted nuclei examined by confocal microscopy. I, interphase; M, mitotic. (b) The oocyte-specific transcription factor TBP2 (red) is taken up by mitotic nuclei to a far greater extent than interphase nuclei by 48 h. GFP labelled histone B4 is used to mark the position of transplanted mitotic and interphase nuclei. The arrows indicate examples of one interphase (yellow) and one mitotic nucleus (white). (TIF) [file pbio.1001914.s003.tif]

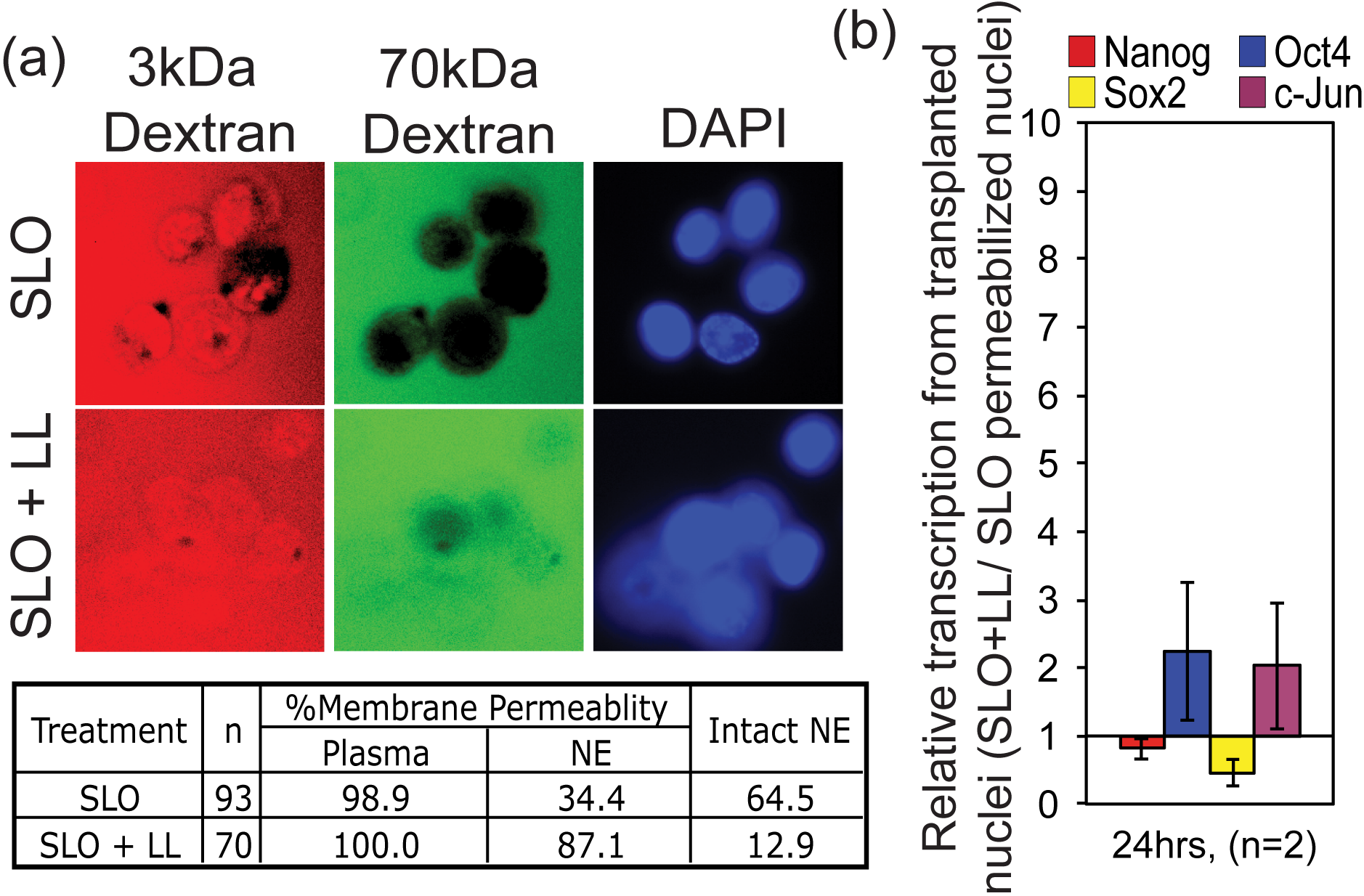

Supplement: Figure S3 — Permeabilization of the nuclear membrane does not reduce mitotic advantage. (a) 3 KDa dextran enters SLO-permeabilized nuclei, but 70 KDa dextran does not (black spheres). SLO and LL together permit entry of 70 KDa dextran nucleoplasm. The table illustrates the proportion of permeabilized plasma and nuclear membranes by these treatments. (b) Transcriptional reprogramming 24 h after transplantation of SLO or SLO+LL permeabilized nuclei to oocyte GVs is similar. Supporting data can be found in Data S1. (TIF) [file pbio.1001914.s004.tif]

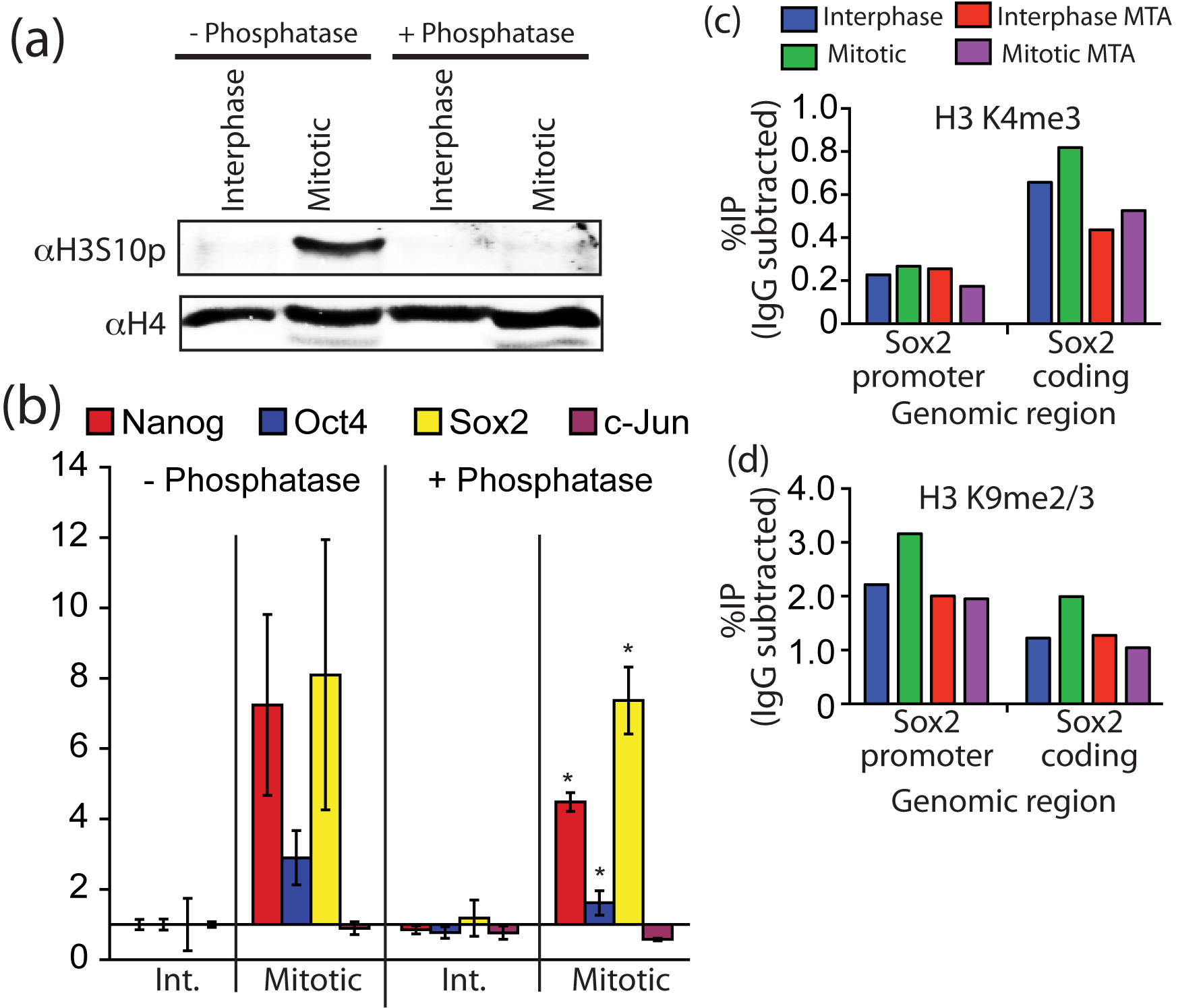

Supplement: Figure S4 — Phosphatase treatment does not decrease mitotic advantage. (a) Phosphatase removes phosphorylation of H3S10 from mitotic nuclei. (b) Phosphatase treatment does not eliminate mitotic advantage. Supporting data can be found in Data S1. (c) MTA may be used to reduce the local methylation of some genes on mitotic entry. ChIP against the Sox2 promoter and coding sequence using an antibody against H2K4me3 and (d) H3K9me2/3. Supporting data can be found in Data S1. (TIF) [file pbio.1001914.s005.tif]
